# The Role of Recombinant Human Bone Morphogenetic Protein–2 in Atlantoaxial Arthrodesis: Institutional Predictors and Systematic Review of Outcomes

**DOI:** 10.3390/jcm14248731

**Published:** 2025-12-10

**Authors:** Mazen Taman, Abigail A. Teshome, Arjun Ganga, Elijah M. Persad, Owen P. Leary, Senay Gokcebel, Rahul Sastry, Patricia Zadnik Sullivan, Ki-Eun Chang, Adetokunbo A. Oyelese, Ziya L. Gokaslan, Jared S. Fridley, Tianyi Niu

**Affiliations:** 1 Department of Neurosurgery, Warren Alpert Medical School of Brown University, Providence, RI 02903, USAjared.fridley@austin.utexas.edu (J.S.F.); tianyi_niu@brown.edu (T.N.); 2 Stanford Health Care, Department of Psychiatry and Behavioral Sciences, Stanford University School of Medicine, Stanford, CA 94305, USA; 3 School of Public Health, Brown University, Providence, RI 02903, USA

**Keywords:** BMP, atlantoaxial instability, spinal fusion outcomes, C1–2 fusion, bone morphogenetic protein-2

## Abstract

**Background/Objectives:** To evaluate the safety, efficacy, and predictors of rhBMP-2 use in atlantoaxial (C1–2) fusion through institutional outcomes and a pooled analysis from the literature. **Methods:** We retrospectively analyzed adult patients who underwent C1–2 fusion at a tertiary care center between 2015 and 2020. Primary outcomes included radiographic fusion and postoperative complications. Univariate logistic regression was used to identify predictors of intraoperative rhBMP-2 use. A subset of eight patients who underwent isolated C1–2 fusion using rhBMP-2 were analyzed and evaluated against 260 cases from a PRISMA-compliant systematic review of the literature. **Results:** Among our 49 patients (mean age, 61.8 ± 22.8 years; 57% female), 24 (49%) received rhBMP-2. Radiographic fusion or early signs were observed in 89.8% of patients, with no significant difference between rhBMP-2 and non-rhBMP-2 groups (*p* = 0.678). Complications were similar (12.5% vs. 25.0%, *p* = 0.702). All postoperative instabilities occurred in non-rhBMP-2 patients (*p* = 0.043). Degenerative pathology was associated with increased use of rhBMP-2 (OR of 7.96, *p* = 0.051), while trauma was associated with decreased use (OR of 0.21, *p* = 0.019). Among the eight local isolated cases, seven achieved fusion and none required revision. Our literature review yielded a group of 260 patients, 241 of which achieved successful fusion with a complication rate of 10.8% and a revision surgery rate of 3.8%. **Conclusions:** rhBMP-2 appears to be a safe and effective adjunct in C1–2 fusion, with outcomes comparable to alternative techniques. Although off-label, its selective use may benefit patients at risk of nonunion. Interpretation should be tempered by the retrospective design and heterogeneity across cohorts; pooled rates are descriptive. Further prospective studies are needed to establish standardized protocols, optimal dosing, and long-term efficacy.

## 1. Introduction

The atlantoaxial (C1–2) joint is the most mobile segment of the spine and accounts for up to 60% of cervical rotation, making it particularly susceptible to instability from trauma, congenital anomalies, neoplasms, inflammatory disease, and degeneration [1,2]. Achieving durable fusion at C1–2 is technically demanding and historically less reliable than that at subaxial levels [3,4,5]. Among stabilization strategies, the Goel-Harms construct, utilizing C1 lateral mass and C2 pedicle screw fixation, offers superior biomechanics relative to posterior wiring and transarticular techniques [6]. However, anatomic variation, proximity to critical neurovascular structures, and prior surgery can complicate fixation and affect outcomes. As such, the optimization of adjunct factors that facilitate bony fusion is of paramount importance in the case of atlantoaxial fusion.

Recombinant human bone morphogenetic protein-2 (rhBMP-2) is a multipotent cell differentiating factor, growth factor, and osteoinductive agent that promotes de novo bone formation even at non-bony sites [7]. Despite broad clinical adoption, its use remains controversial due to reports of wound problems, heterotopic ossification, radiculopathy, increased swelling with subsequent dysphagia (e.g., retropharyngeal and anterior), and potential cancer risks [8,9,10,11,12]. Since its 2002 approval, rhBMP-2 has been FDA-approved only for anterior lumbar interbody fusion, with a black box warning for cervical spine swelling and potential fatal outcomes [13]. Consequently, about 85% of rhBMP-2 usage is off-label, including in cervical spinal fusions [14]. Moreover, there is no consensus on appropriate dosing and preoperative indications [13].

Evidence supporting rhBMP-2 largely derives from lumbar fusion cohorts, often 1–2 level degenerative cases [15]. By contrast, data specific to atlantoaxial fusion are limited and there is no consensus regarding indications, dose, or technique at C1–2, despite the joint’s unique biomechanical demands and the high clinical cost of nonunion. This gap constrains decision-making, particularly in patients at elevated risk of pseudarthrosis or when structural graft options are limited.

To address this, we combined a single-center retrospective cohort of C1–2 fusions with a PRISMA-guided systematic review focused on isolated atlantoaxial arthrodesis using rhBMP-2. We sought to (1) describe fusion and complication outcomes, (2) identify clinical predictors of intraoperative rhBMP-2 use, and (3) contextualize institutional results within pooled literature to inform practical, dose-conscious application at C1–2.

## 2. Materials and Methods

### 2.1. Local Case Series

We conducted a single-center retrospective cohort study of adult patients who underwent atlantoaxial fusion at a tertiary care institution between August 2015 and December 2020. Inclusion criteria were: (1) posterior cervical or occipitocervical fusion involving the C1–2 segment, (2) at least three months of clinical follow-up, and (3) available postoperative imaging. Pediatric patients and cases performed for tumor-related instability were excluded.

Demographic, clinical, and operative variables were abstracted, including age, sex, surgical indication, prior cervical surgery, levels fused, use of decompression, and adjunct fusion materials. Etiology was classified a priori as traumatic, degenerative, or congenital/deformity based on operative indication. The primary exposure was intraoperative use of rhBMP-2. Outcomes included radiographic arthrodesis, atlantoaxial stability, and postoperative complications (e.g., instrumentation failure, pseudoarthrosis, wound complications, reoperation, infection). Our primary criterion was CT evidence of continuous bony bridging across the intended C1–2 arthrodesis bed without intervening lucency. When CT was not available, fusion was defined as stability on flexion–extension radiographs with no motion across C1–2 and no hardware lucency, as documented by a board-certified radiologist. Early fusion was recorded if imaging occurred before six months.

Instability was assessed using flexion–extension radiographs when clinically indicated. Prespecified complication definitions were: wound complication (dehiscence, deep/organ-space infection, or return to the operating room for wound-related care), airway/soft-tissue edema (symptomatic compressive collection requiring steroids, observation, or procedure), neurologic event (new focal deficit), and instrumentation failure (any postoperative event reflecting mechanical compromise of the construct, including screw or rod breakage, screw loosening with loss of cortical engagement, or hardware migration on imaging, irrespective of fusion status). The need for reoperation and clinical sequelae were separately recorded.

Follow-up was retrospective and non-protocolized. Patients were typically seen at ~6 weeks, 3–6 months, and ~12 months. Assessment began with flexion–extension radiographs; CT was obtained when radiographs were equivocal, symptoms evolved, or per surgeon preference. ‘Time to fusion’ was defined as the date of the earliest postoperative study that met our fusion definition.

All surgeries were performed using a posterior approach by fellowship-trained spine neurosurgeons, typically with resident participation. In all rhBMP-2 cases, the collagen sponge was cut to size and confined to the C1–2 joint space or laid over decorticated posterior elements, with deliberate avoidance of the spinal canal and paraspinal soft tissues. Hemostasis and a watertight layered closure were emphasized to mitigate cervical-specific risks of postoperative swelling or fluid collection. Adjunct demineralized bone matrix (DBX) and/or local autograft were used at the discretion of the operating surgeon. Commercial kit sizes were recorded and converted to total rhBMP-2 mass according to manufacturer fill (XX-small ≈ 1.05 mg, X-small ≈ 2.1 mg, small ≈ 4.2 mg).

Statistical analyses were performed in Stata 15 (StataCorp, College Station, TX, USA) and R version 4.1.1 (R Foundation for Statistical Computing, Vienna, Austria). Continuous variables were compared with Wilcoxon rank-sum or Kruskal–Wallis test, and categorical variables with Fisher’s exact test (two-sided, α = 0.05). Univariate logistic regression identified predictors of rhBMP-2 use. Multivariable logistic models assessed (i) predictors of intraoperative rhBMP-2 use and (ii) associations of rhBMP-2 with fusion and postoperative adverse events. Covariates included surgical indication, sex, and number of levels fused. Among rhBMP-2 recipients, we performed a dose-stratified analysis (≤1.05 mg, 1.05–2.10 mg, >2.10 mg) comparing fusion and complication rates across dose bins. This study was approved by the Institutional Review Board (protocol #816619) with a waiver of informed consent.

### 2.2. Systematic Literature Review

A systematic review was conducted following Preferred Reporting Items for Systematic Reviews and Meta-Analyses (PRISMA) guidelines [16]. Pubmed, Embase, and Cochrane databases were searched through November 1, 2022, using the terms: ((bone morphogenetic protein) OR (BMP)) AND ((atlantoaxial) OR (C1–2) OR (C1–C2)) AND ((spine) OR (fusion)) (File S1).

Two investigators independently screened titles, abstracts, and full texts using Covidence (Veritas Health Innovation, Melbourne, Australia). Discrepancies were resolved by consensus with a third reviewer. Only adult patients undergoing C1–2 fusion with rhBMP-2 were included; cases involving subaxial or occipitocervical constructs without isolated atlantoaxial outcomes were excluded. Risk of bias was assessed using the Cochrane ROBINS-I tool across seven domains: confounding, patient selection, intervention classification, deviations from intended interventions, missing data, outcome measurement, and selective reporting [17]. Studies were rated as low, moderate, serious, or critical risk of bias.

Data extracted included study characteristics (author, year, design), patient demographics, rhBMP-2 dose and delivery method, fusion assessment criteria, complications, revision surgeries, and adjunct graft materials.

Institutional isolated C1–2 cases were then pooled with eligible published series to calculate overall fusion, complication, and revision rates. Descriptive statistics were generated in Microsoft Excel (Version 2016; Microsoft, Redmond, WA, USA) and Stata SE.

## 3. Results

### 3.1. Local Cohort: Patient Characteristics and Outcomes

From 2015 to 2020, 65 patients underwent atlantoaxial fusion at our institution. After excluding pediatric patients (*n* = 4), cases without follow-up (*n* = 7), and tumor-related instability (*n* = 5), 49 adults were included (Table 1). Of these, 24 (49.0%) received rhBMP-2 at the C1–2 level.

**Figure 1:**
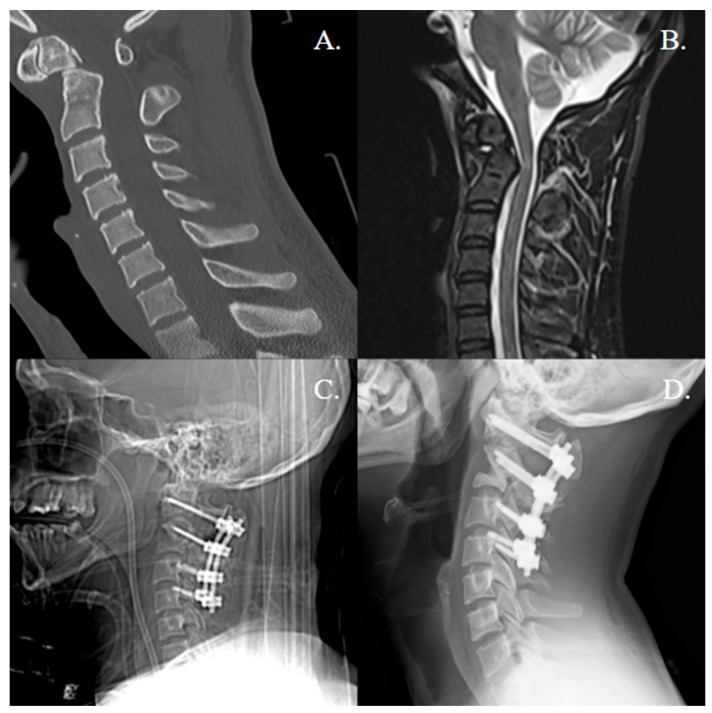
Odontoid fracture (motor-vehicle collision): pre-op imaging, construct, and fusion. (**A**) Sagittal cervical CT showing a Type II odontoid fracture at the base of the dens. (**B**) Pre-operative sagittal T2-weighted MRI demonstrating posterior protrusion of the fractured odontoid into the spinal canal without cord signal change. (**C**) Immediate post-operative lateral radiograph showing C1 lateral mass and C2 pedicle/translaminar screws connected with rods, with appropriate reduction and hardware position. (**D**) One-year post-operative lateral radiograph demonstrating maintained alignment, and no hardware lucency, consistent with stable arthrodesis.

**Figure 2:**
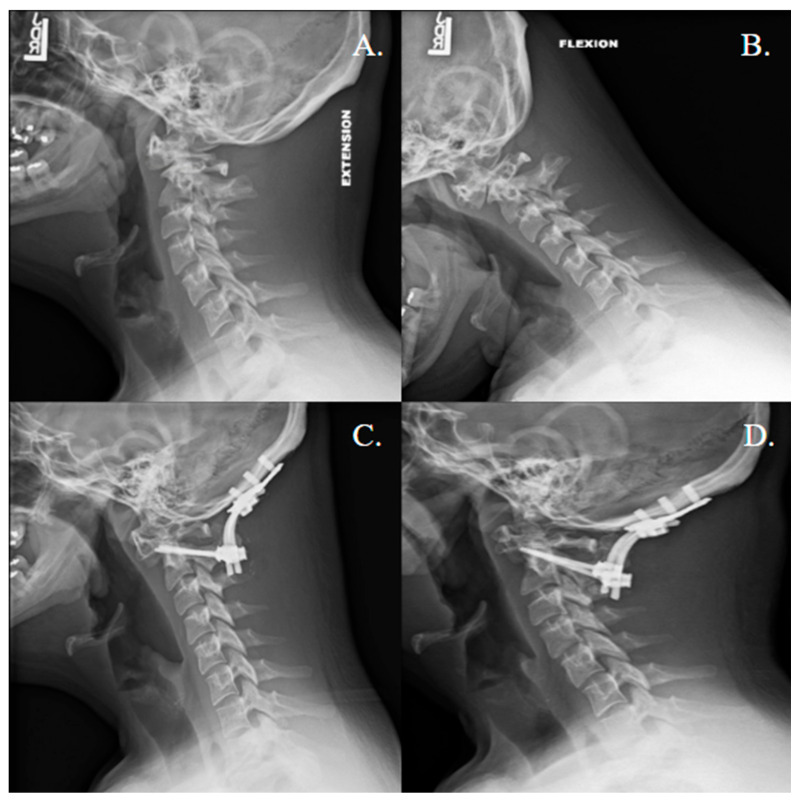
Atlantoaxial instability in Down syndrome: dynamic instability, construct, and follow-up. (**A**) Pre-operative lateral extension radiograph with ADI measuring 3 mm. (**B**) Pre-operative lateral flexion radiograph with anterior ADI measuring 6 mm, demonstrating dynamic C1–2 instability. (**C**) Immediate post-operative lateral radiograph demonstrating C1–2 instrumented fusion with appropriate hardware position and reduction in the ADI. (**D**) One-year post-operative lateral radiograph showing maintained reduction, no hardware lucency, consistent with fusion. Abbreviations: ADI: anterior antlanto dental interval; LDR: low dose reconstruction.

The mean age was 61.8 years (SD ± 22.8), with 28 females (57.1%) and 21 males (42.9%). Surgical indications included trauma (63.3%), degenerative disease (24.5%), and congenital anomalies (12.2%) (Figure 1 and Figure 2). Concomitant decompression was performed in 59.2% of cases, and 4.1% had prior cervical surgery. Across the literature, patients with degenerative pathology were more likely to receive rhBMP-2, though these findings were not significant (*p* = 0.051). Among cases receiving rhBMP-2, the median total dose was 1.05 mg (interquartile range 1.05–2.10 mg; range 1.05–4.20 mg). Doses corresponded to 16 XX-small kits (66.7%), 4 X-small kits (16.7%), and 4 small kits (16.7%).

Mean follow-up was 15.7 months (17.9 months in rhBMP-2 vs. 13.5 months in non-rhBMP-2, *p* = 0.364). Overall, 44 patients (89.8%) demonstrated radiographic fusion or early signs of fusion, with no significant difference between rhBMP-2 and non-rhBMP-2 groups (*p* = 0.678). Time to fusion varied across patients. Recurrent features among delayed fusion included low bone quality/osteoporosis, smoking, diabetes, chronic corticosteroid use, longer constructs or concomitant decompression, limited autograft (greater allograft/DBC), and early postoperative NSAID exposure; no single factor reached statistical significance in this cohort. Fusion was assessed by CT in 24/49 (49.0%) and by dynamic radiographs in 25/49 (51%) of local cases. Among rhBMP-2 cases, fusion did not differ by dose bin (≤1.05 mg: 15/16 [93.8%]; 1.05–2.10 mg: 3/3 [100%]; >2.10 mg: 4/4 [100%]; *p* = 1.00).

Eight patients (16.3%) experienced complications, most commonly, instrumentation failure (*n* = 4). Two of which occurred in the rhBMP-2 group and two in the non-rhBMP-2 group. Of the failures in the rhBMP-2 group, one involved bilateral C1 screw breakage discovered after a motor-vehicle collision; imaging demonstrated solid C1–2 arthrodesis and no reoperation was required. The other was an early postoperative screw issue that was revised the following day without lasting deficit. In the non-rhBMP-2 group, one case underwent reoperation for hardware loosening, and another had planned reoperation for suspected loosening; neither case was associated with neurologic decline at last follow-up. Across all failures, there was no mortality and no case of symptomatic nonunion attributable to the failure. All cases of postoperative instability, distinct from hardware events, occurred in the non-rhBMP-2 group (*n* = 4; *p* = 0.043).

Complication and reoperation rates were similar between groups. Notably, there were no cases of symptomatic postoperative compressive fluid collection, airway edema, or dysphagia attributable to rhBMP-2, and no reoperations for seroma or hematoma. These findings were unchanged in the subset that underwent posterior decompression with thecal sac exposure. Among rhBMP-2 cases, complication rates were 6.3% (≤1.05 mg), 66.7% (1.05–2.10 mg), and 0% (>2.10 mg), respectively (*p* = 0.047).

### 3.2. Predictors of rhBMP-2 Use

Univariate logistic regression identified degenerative pathology as a strong predictor of rhBMP-2 use (OR of 7.96; 95% CI of 1.45–62.62; *p* = 0.051), while trauma was associated with reduced likelihood of use (OR of 0.21; 95% CI of 0.06–0.72; *p* = 0.016) (Table 2). Age, sex, number of fused levels, prior surgery, and decompression did not significantly influence rhBMP-2 application (all *p* > 0.05). After adjustment, degenerative indication trended toward greater rhBMP-2 use and trauma toward less, but no predictor reached significance (all *p* > 0.05). rhBMP-2 use was not associated with higher odds of complications and showed a nonsignificant trend toward higher odds of fusion.

### 3.3. Isolated Atlantoaxial Fusion Cases

Among patients receiving rhBMP-2, eight (33.3%) underwent isolated C1–2 fusion (Table 3). Mean age was 53.7 years (range 28–80), and most were treated for trauma (87.5%). Follow-up averaged 18.4 months, with radiographic assessment available at 12.4 months.

All patients received rhBMP-2–soaked collagen sponges placed intra-articularly, often with adjuncts such as DBX, morselized autograft, or femoral head allograft. Seven of eight patients (87.5%) achieved fusion or early fusion signs (Table 4). Imaging modality was evenly split (4/8 CT, 4/8 radiographs). One patient experienced C1 screw breakage at 20 months but did not require revision due to solid arthrodesis. No cases of postoperative instability or neurologic decline were observed.

### 3.4. Systematic Literature Review and Pooled Analysis

**Figure 3:**
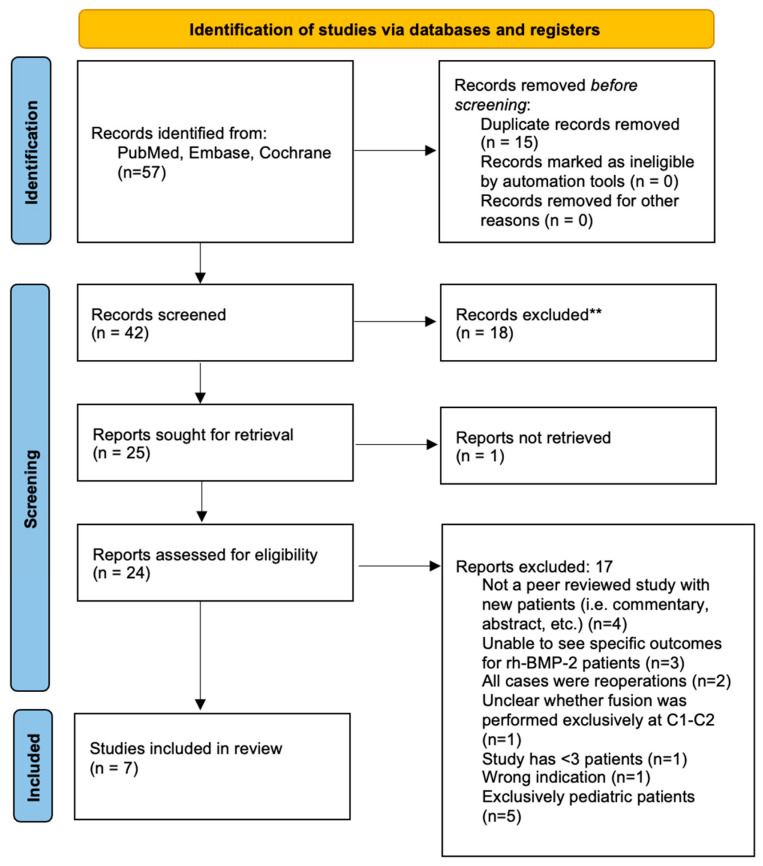
PRISMA flow diagram highlighting C1–2 fusion cases treated with rhBMP–2 in the literature. (** Records were excluded for pediatric cases and cases involving multi-level constructs).

The database search yielded 57 unique records (Figure 3); seven studies met inclusion criteria, comprising 260 patients treated with rhBMP-2 at C1–2 (Table 5) [18,19,20,21,22,23,24,25,26,27,28]. Fusion criteria varied, including plain radiograph, dynamic radiograph, and CT-confirmed bridging and Lenke classification [29].

Fusion was achieved in 92.7% (241/260) of cases with available follow-up. The overall complication rate was 10.8% (28/260) and the revision rate was 3.8% (10/260). Reported complications included instrumentation failure (*n* = 7), wound-healing issues (*n* = 6), pseudoarthrosis (*n* = 5), adjacent segment instability (*n* = 3), and dural tear (*n* = 2).

## 4. Discussion

Achieving reliable arthrodesis at the atlantoaxial joint remains challenging due to its high mobility and complex anatomy. Our study represents the largest combined dataset to date evaluating rhBMP-2 specifically at C1–2. By integrating a single-institution cohort with a systematic review, we provide a more comprehensive assessment of the safety, efficacy, and patterns of rhBMP-2 use in this anatomically demanding region [31].

In this cohort, rhBMP-2 was applied in small, well-contained doses at C1–2, with a median of 1.05 mg and an upper bound of 4.2 mg. Within this dosing range, fusion and complication profiles were comparable to cases without rhBMP-2. Notably, instrumentation failures were infrequent and balanced between groups; the single post-collision screw breakage occurred in the setting of a solid arthrodesis and did not require reoperation. These observations support that judicious, low-dose rhBMP-2 use at C1–2 can be incorporated without excess mechanical or clinical morbidity.

The off-label use of rhBMP-2 in cervical fusion has long been debated. Iliac crest autograft remains the gold standard, but limitations in availability and donor site morbidity have encouraged use of biologic substitutes [32,33]. Additionally, there is no consensus on the appropriate dosing of rhBMP-2, and higher doses have been linked to increased rates of wound-related complications, infections, and neurological deficits [34,35,36,37,38]. A systematic review by Hofstetter et al. involving 5890 patients found significant variability in dosing [34]. Furthermore, although rhBMP-2 is FDA-approved for anterior lumbar interbody fusion, cervical applications remain off-label due to concerns about postoperative swelling, wound complications, dysphagia, and potentially increased cancer risk [8,9,10,11,12,13,14,34]. Posterior cervical fusion carries unique complications compared to lumbar fusion, including arterial injury (particularly vertebral artery damage), dural injury, implant failure, infection, thromboembolism, and instrumentation malfunction [39,40,41]. In a 69-patient series of posterior C1–2 arthrodesis using intra-articular rhBMP-2 without structural graft, Ishida et al. reported a 10.1% instrumentation failure rate but no ectopic bone formation or soft-tissue edema [30]. Despite these concerns, our findings support that when used in small, well-contained doses during posterior C1–2 arthrodesis, rhBMP-2 can achieve high rates of fusion with acceptably low complication and revision rates.

While concerns about cervical rhBMP-2 center on postoperative soft-tissue swelling and compressive collections, our posterior C1–2 cohort showed no signal for these events. Complications and unplanned returns to the operating room did not differ between groups, and even in cases with thecal sac exposure there were no compressive collections requiring intervention. These data suggest that, when dosing is conservative and placement is confined, posterior C1–2 application can avoid the complications that motivated the black box warning in anterior cervical settings.

In our institutional cohort, nearly half the patients received rhBMP-2. Fusion was observed in 91.7% of rhBMP-2 cases, consistent with published series. There were no statistically significant differences in fusion or complication rates between rhBMP-2 and non-rhBMP-2 groups. Dose-stratified analyses within our cohort did not show a relationship between dose and fusion. An apparent difference in complication proportions across bins was driven by very small, unbalanced bins (*n* = 3–16). These findings should be interpreted as exploratory rather than establishing an optimal dose. All four cases of postoperative instability occurred in the non-rhBMP-2 group; however, given the retrospective design and non-standardized technique, levels fused, graft choice, and postoperative care, we do not infer a definitive protective effect. Taken together, judicious rhBMP-2 use at C1–2 appeared safe in our series and was associated with high fusion rates.

When we restricted our analysis to isolated C1–2 fusions, both institutional and published data revealed favorable outcomes. Across 268 total cases, including 8 from our center and 260 from the literature, fusion was achieved in 92.5% of patients, with complication and revision rates of 10.8% and 3.7%, respectively. These findings are particularly meaningful given the relatively high nonunion rates traditionally observed at the C1–2 junction [3,4,5]. Although, it is important to note that heterogeneity in operative technique and study design should be considered when analyzing these groups together.

Predictors of rhBMP-2 use also reflected clinical context. Degenerative pathology was strongly associated with rhBMP-2 application, while trauma cases were less likely to receive it, likely reflecting surgeon caution in acute settings due to theoretical risks of ectopic bone formation or neurologic compromise. Nonetheless, seven of eight isolated trauma cases at our institution treated with rhBMP-2 achieved fusion, underscoring its potential role in post-traumatic instability when carefully applied.

rhBMP-2 carries a meaningful direct implant cost that varies by kit size and dose. Value should be considered alongside indirect costs that differ by strategy. Iliac crest harvest can add donor-site morbidity and analgesic needs. Operative time, length of stay, readmissions, and the risk of nonunion with possible reoperation also drive cost. Our dataset did not include charges, resource use, or quality-of-life measures, so we did not perform a cost-effectiveness analysis. Future studies should prospectively capture implant cost by dose, operative time, length of stay, 90-day readmissions, reoperations, and patient-reported outcomes to enable cost-utility modeling.

Several comparative studies add perspective. Yan et al. reported similar fusion and complication rates between rhBMP-2 and iliac crest autograft in older adults, while Guppy et al. and Hood et al. reported 100% fusion without revisions in their rhBMP-2 cohorts [20,23,28]. Pooled across studies, rhBMP-2 appeared to outperform non-rhBMP-2 techniques in fusion rates (90% vs. 82%, *p* = 0.04), with no significant difference in complication (7% vs. 5%, *p* = 0.480) or revision (2.2% vs. 0%, *p* = 0.089) rates. Because complication definitions and causal attribution to rhBMP-2 vary across studies, we interpret pooled proportions as descriptive experience rather than comparative risk estimates. Within our institution, prespecified definitions were applied uniformly, but cross-study heterogeneity limits causal inference. Overall, rhBMP-2 remains a potentially valuable adjunct; its cost should be weighed against the clinical and economic impact of preventing nonunion and revision in appropriately selected patients.

### Limitations

This study has several limitations. First, our institutional analysis was retrospective, with variable surgical protocols and no standardized dosing or fusion assessment. Radiographic follow-up was inconsistent, fusion was sometimes inferred from radiology reports rather than uniform CT, and non-uniform imaging intervals make fusion timing imprecise and prone to misclassification. Second, although our literature review followed PRISMA methodology, included studies were heterogeneous in design, risk of bias, and outcome definitions. Consequently, pooled proportions summarize observed experience rather than estimate causal effects, and the overall complication rate may be influenced by publication and selective-reporting biases. Finally, the small number of isolated C1–2 cases limits statistical power, and regression models are susceptible to wide confidence intervals and potential overfitting. As such, our sample was underpowered to identify determinants of delayed fusion; observed contributors are hypothesis-generating.

Additionally, procedures were performed by multiple fellowship-trained surgeons, introducing selection/performance bias along with significant heterogeneity in graft selection, dosing, and perioperative management. Nearly all rhBMP-2 cases involved concomitant use of autograft, allograft, or demineralized bone matrix, making it difficult to isolate the independent effect of rhBMP-2. Moreover, dosing and placement techniques were inconsistently reported, and fusion criteria varied across studies. The observed imbalance in postoperative instability, limited to non-rhBMP-2 cases, should be considered hypothesis-generating, as unmeasured differences in case mix, technique, and biologic selection could account for this pattern. Together, these factors constrain direct comparisons and highlight the need for standardized protocols with newer biologics such as cellular bone matrices and synthetic bone graft substitutes. A multicenter prospective study or registry with standardized dosing, fusion metrics, and long-term outcomes would provide critical insight into the safety and cost–benefit profile of rhBMP-2 in upper cervical fusion.

Despite these limitations, our findings support the selective, judicious use of rhBMP-2 in atlantoaxial fusion, particularly in patients at risk of nonunion or when graft options are limited.

## 5. Conclusions

rhBMP-2 appears to be a safe and effective adjunct for atlantoaxial fusion, with high fusion rates and low complication rates in both institutional and published series. Although its use in the cervical spine remains off-label, our findings support selective application at C1–2, particularly in patients at elevated risk of nonunion. Continued prospective research with standardized dosing, technique, and outcome reporting is needed to define its role more clearly and to optimize cost-effectiveness in this challenging region.

## Figures and Tables

**Table 1:** Baseline patient demographics, surgical characteristics, and outcomes for local C1–2 arthrodesis cases stratified by rhBMP-2 use.

	Non-rhBMP-2 Group	rhBMP-2 Group	Overall	*p*-Value
	(N = 25)	(N = 24)	(N = 49)	
Age at Surgery: Mean (SD)	66.85 (18.47)	56.56 (25.84)	61.82 (22.75)	0.118
Gender:				0.333
Male: N (%)	21 (84.0)	12 (50.0)	28 (57.14)	
Female: N (%)	16 (64.0)	12 (50.0)	21 (42.86)	
Fusion Indication:				
Traumatic: N (%)	20 (80.0)	11 (45.83)	31 (63.27)	0.019
Congenital/Deformity: N (%)	2 (8.0)	4 (16.67)	6 (12.24)	0.417
Degenerative: N (%)	3 (12.0)	9 (37.50)	12 (24.49)	0.051
Prior Cervical Surgery:				0.977
Yes (%)	1 (4.0)	1 (4.17)	2 (4.08)	
No (%)	21 (96.0)	23 (95.83)	47 (95.92)	
Number of Levels Fused: Mean (SD)	3.76 (1.85)	3.58 (1.69)	3.67 (1.76)	0.729
Atlantoaxial Fusion Only:				0.481
Yes (%)	6 (24.0)	8 (33.33)	14 (28.57)	
No (%)	19 (76.0)	16 (66.67)	35 (71.43)	
Concomitant Decompression:				0.306
Yes (%)	16 (64.0)	13 (54.17)	29 (59.18)	
No (%)	9 (36.0)	11 (45.83)	20 (40.82)	
Number of Levels Decompressed:	1.72 (2.07)	2.21 (1.98)	1.96 (2.02)	0.403
Mean (SD)				
Follow-up Time (Months):	13.52 (11.72)	17.88 (20.19)	15.65 (16.40)	0.364
Mean (SD)				
Pain Reported Post-Operatively:				0.467
Yes (%)	5 (20.0)	7 (29.17)	12 (24.49)	
No (%)	20 (80.0)	17 (70.83)	37 (75.51)	
Fusion or Early Signs on Post-Op				0.678
Imaging:				
Yes (%)	22 (88.0)	22 (91.67)	44 (89.80)	
No (%)	3 (12.0)	2 (8.33)	5 (20.10)	
Post-Operative Imaging Used:				0.467
X-Ray: N (%)	12 (48.0)	13 (52.0)	25 (51.02)	
CT: N (%)	13 (54.17)	11 (45.83)	24 (49.0)	
Post-Operative Instability:				0.043
Yes (%)	4 (16.0)	0 (0)	4 (8.16)	
No (%)	21 (84.0)	24 (100)	45 (91.84)	
Complications:				0.702
Yes (%)	5 (25.0)	3 (12.5)	8 (16.33)	
No (%)	20 (75.0)	21 (87.5)	41 (83.67)	
Reoperation:				0.977
Yes (%)	1 (4.0)	1 (4.17)	2 (4.08)	
No (%)	24 (96.0)	23 (95.83)	47 (95.52)	
Pseudoarthrosis:				0.327
Yes (%)	1 (4.0)	0 (0.0)	1 (2.04)	
No (%)	24 (96.0)	24 (100.0)	48 (97.96)	
Instrument Breakage/Hardware				1.000
Malfunction				
Yes (%)	2 (8.0)	2 (8.33)	4 (8.16)	
No (%)	23 (92.0)	22 (91.67)	45 (91.84)	
Wound Complication:				0.327
Yes (%)	1 (4.0)	0 (0.0)	1 (2.04)	
No (%)	24 (96.0)	24 (100.0)	48 (97.96)	

**Table 2:** Odds or receiving rhBMP-2 on selected, pre-operative clinical variables.

	Odds of Receiving rhBMP-2 (95% CI)	*p*-Value
Age	0.98 (0.927–1.011)	0.168
Gender	0.42 (0.101–1.595)	0.215
Number of levels fused	0.87 (0.547–1.331)	0.542
Degenerative indication	7.96 (1.452–62.619)	0.027
Congenital indication	0.98 (0.054–17.609)	0.990
Traumatic indication	0.21 (0.055–0.717)	0.016
Concurrent decompression	1.92 (0.388–10.41)	0.425
Prior surgery	0.721 (0.025–20.980)	0.830

**Table 3:** Characteristics of isolated C1–C2 rhBMP-2 cases.

ID	Age, Sex	Indication	Construct	Concurrent C1–2 Decompression (Y/N)	Arthrodesis Material	Follow Up (Months)
1	45, M	Degenerative	C1 lateral mass screws bilaterally, C2 translaminar screws bilaterally, titanium rod	N	DBX, rhBMP-2 (XX small kit, 0.82 mg/mL)	26
2	29, F	Trauma	C1 lateral mass screws bilaterally, C2 translaminar screws bilaterally. Sublaminar Songer cables at C1 and posterior to C2 spinous process, titanium rod	N	DBX, femoral head structural allograft, rhBMP-2 (XX small kit, 0.82 mg/mL)	25
3	51, M	Trauma	C1 lateral mass screws bilaterally, 1 C2 pars and 1 C2 pedicle screw, titanium rods	N	rhBMP-2 (X small kit, 1.62 mg/mL)	3
4	28, F	Trauma	C1 lateral mass screws bilaterally, C2 pedicle screws bilaterally, titanium rod	N	DBX, local autograft, rh-BMP-2 (half of a XX small kit, 0.41 mg/mL)	20
5	80, F	Trauma	C1 lateral mass screws bilaterally, C2 pedicle screws bilaterally, titanium rod, cross connector at C1	N	DBX, local autograft, rhBMP-2 (X small kit, 1.62 mg/mL)	4
6	33, F	Trauma	C1 lateral mass screws bilaterally, C2 pedicle screws bilaterally, titanium rod	N	DBX, local autograft, rhBMP-2 (half of a XX small, 0.41 mg/mL)	69
7	77, M	Trauma	C1 lateral mass screws bilaterally, C2 pedicle screws bilaterally	Y	DBX, local autograft, rh-BMP-2 (XX small kit, 0.82 mg/mL)	10
8	80, M	Trauma	C1 lateral mass screws bilaterally, C2 pedicle screws bilaterally, titanium rods	Y	DBX, local autograft, rh-BMP-2 (XX small kit, 0.82 mg/mL)	3

**Table 4:** Postoperative outcomes of Local isolated C1–C2 cases.

ID	Surgical Complications	Revision (Y/N)	Fusion on Latest Follow-Up Imaging (Y/N)	Atlantoaxial Instability Clinically or in Dynamic Imaging? (Y/N)	Imaging Follow Up (Time, Type)
1	C1 screw breakage	N, avoided re-op due to complete bony fusion	Y	N	25 months, X-ray
2	None	N	Y	N	25 months, X-ray
3	None	N	Early signs of fusion	N	3 months, CT
4	None	N	Y	N	19 months, CT
5	None	N	N	N	4 months, CT
6	None	N	Y	N	15 months, X-ray
7	None	N	Early signs of fusion	N	3 months, CT
8	None	N	Early signs of fusion	N	3 months, X-ray

**Table 5:** C1–C2 arthrodesis cases in the literature.

Author, Year (*Journal*)	Number of Patients	Mean rhBMP-2 Dose (mg)	ROBINS-1 Risk of Bias	Fusion Definition	Successful Fusion	Complications	Revision Surgeries	Other Fusion Materials Used
Guppy et al., 2019 * (*World* *Neurosurgery*) [20]	58	unreported	Low	Lack of symptomatic nonunion	58/58	0/58	0/58	N/a
Hamilton et al., 2010 (*Journal of Craniovertebral Junction and Spine*) [21]	7	2.38	Moderate	Lenke classification assessed on CT	7/7	0/7	0/7	Morselized allograft
Hood et al., 2014 (*World* *Neurosurgery*) [23]	52	4.5	Low	Lenke classification assessed on plain radiograph and CT	50/50 (2 patients lost during follow-up)	0/50	0/50	Cancellous allograft (20 patients) Iiliac allograft (24 patients)
Siemionow et al., 2017 * (*J Neurol Surg A Cent Euro Neurosurgery*) [26]	3	4	Moderate	N/a	2/3	2/3 (adjacent segment instability + nonunion in 1 patient, pseudoarthrosis in other patients)	1/3 (pseudoarthrosis and progressive instability)	Allograft (unspecified, 2 patients) Iliac crest autograft (1 patient)
Traynelis et al., 2021 (*JNS Spine*) [27]	3	N/a	Low	No motion on dynamic radiographs, intact hardware, and no lucencies around the graft	3/3	N/a	N/a	Allograft (unspecified, 3)
Yan et al., 2014 * (*Orthopedics*) [28]	68	N/a	Moderate	Lack or presence of fusion on post-operative CT scan	56/68	7/68 (wound complications in 6 patients, dural tear in 1 patient)	2/68 (wound-related)	Iliac crest bone graft (all patients)
Ishida et al. 2019 (*World* *Neurosurgery*) [30]	69	2.5	Moderate	Stability on plain radiograph, dynamic radiographs, and/or CT scan	65/69	19/69 (1 postoperative difficulty in phonation, 1 incidental durotomy, 2 adjacent segment disease,	7/69 (instrumentation breakage)	13/69 hydroxyapatite 17/69 local autograft chips 24/69 local autograft and allograft chips
Total	260	3.32 across reported studies	-	-	241/260 (92.7%)	2 postoperative dysphagia,	10/260 (3.8%)	-

* Comparative studies; results of control rh-BMP-2 group reported.

## Data Availability

The original contributions presented in this study are included in the article. Further inquiries can be directed to the corresponding author.

## Supplementary Materials

The following supporting information can be downloaded at: https://www.mdpi.com/article/10.3390/jcm14248731/s1, File S1: PRISMA checklist.

## References

[1] Menezes A.H., Traynelis V.C. (2008). Anatomy and biomechanics of normal craniovertebral junction (a) and biomechanics of stabilization (b). Child’s Nerv. Syst..

[2] Yang S.Y., Boniello A.J., Poorman C.E., Chang A.L., Wang S., Passias P.G. (2014). A review of the diagnosis and treatment of atlantoaxial dislocations. Glob. Spine J..

[3] Zhou X., Li S., Liu H., Guo Q., Guo X., Chen F., Han Z., Ni B. (2020). Comparison of Two Bone Grafting Techniques Applied During Posterior C1–C2 Screw-Rod Fixation and Fusion for Treating Reducible Atlantoaxial Dislocation. World Neurosurg..

[4] Yang D.S., Patel S.A., DiSilvestro K.J., Li N.Y., Daniels A.H. (2020). Postoperative complication rates and hazards-model survival analysis of revision surgery following occipitocervical and atlanto-axial fusion. N. Am. Spine Soc. J..

[5] Liu D.D., Rivera-Lane K., Leary O.P., Pertsch N.J., Niu T., Camara-Quintana J.Q., A Oyelese A., Fridley J.S., Gokaslan Z.L. (2021). Supplementation of Screw-Rod C1–C2 Fixation with Posterior Arch Femoral Head Allograft Strut. Oper Neurosurg..

[6] Du Y.-Q., Li T., Ma C., Qiao G.-Y., Yin Y.-H., Yu X.-G. (2020). Biomechanical evaluation of two alternative techniques to the Goel-Harms technique for atlantoaxial fixation: C1 lateral mass–C2 bicortical translaminar screw fixation and C1 lateral mass–C2/3 transarticular screw fixation. J. Neurosurg. Spine.

[7] Subach B.R., Haid R.W., Rodts G.E., Kaiser M.G. (2001). Bone morphogenetic protein in spinal fusion: Overview and clinical update. Neurosurg. Focus.

[8] Faundez A., Tournier C., Garcia M., Aunoble S., Le Huec J.-C. (2016). Bone morphogenetic protein use in spine surgery—Complications and outcomes: A systematic review. Int. Orthop..

[9] Molinari R.W., Kerr C., Kerr D. (2016). Bone morphogenetic protein in pediatric spine fusion surgery. J. Spine Surg..

[10] Epstein N. (2013). Complications due to the use of BMP/INFUSE in spine surgery: The evidence continues to mount. Surg. Neurol. Int..

[11] Perri B., Cooper M., Lauryssen C., Anand N. (2007). Adverse swelling associated with use of rh-BMP-2 in anterior cervical discectomy and fusion: A case study. Spine J..

[12] Smucker J.D., Rhee J.M., Singh K., Yoon S.T., Heller J.G. (2006). Increased swelling complications associated with off-label usage of rhBMP-2 in the anterior cervical spine. Spine.

[13] Hustedt J.W., Blizzard D.J. (2014). The Controversy Surrounding Bone Morphogenetic Proteins in the Spine: A Review of Current Re-search. Yale J. Biol. Med..

[14] Ong K.L., Villarraga M.L., Lau E., Carreon L.Y.M., Kurtz S.M., Glassman S.D. (2010). Off-label use of bone morphogenetic proteins in the United States using administrative data. Spine.

[15] Bannwarth M., Smith J.S., Bess S., Klineberg E.O., Ames C.P., Mundis G.M., Kim H.J., Lafage R., Gupta M.C., Burton D.C. (2021). Use of rhBMP-2 for adult spinal deformity surgery: Patterns of usage and changes over the past decade. Neurosurg. Focus.

[16] Page M.J., McKenzie J.E., Bossuyt P.M., Boutron I., Hoffmann T.C., Mulrow C.D., Shamseer L., Tetzlaff J.M., Akl E.A., Brennan S.E. (2021). The PRISMA 2020 statement: An updated guideline for reporting systematic reviews. BMJ.

[17] ROBINS-I Tool|Cochrane Methods. https://methods.cochrane.org/methods-cochrane/robins-i-tool.

[18] Brockmeyer D.L., Sivakumar W., Mazur M.D., Sayama C.M., Goldstein H.E., Lew S.M., Hankinson T.C., Anderson R.C., Jea A., Aldana P.R. (2018). Identifying Factors Predictive of Atlantoaxial Fusion Failure in Pediatric Patients: Lessons Learned From a Retrospective Pediatric Craniocervical Society Study. Spine.

[19] Desai R., Stevenson C.B., Crawford A.H., Durrani A.A., Mangano F.T.D. (2010). C-1 lateral mass screw fixation in children with atlantoaxial instability: Case series and technical report. J. Spinal Disord. Tech..

[20] Guppy K.H., Lee D.J., Harris J., Brara H.S. (2019). Reoperation for Symptomatic Nonunions in Atlantoaxial (C1–C2) Fusions with and without Bone Morphogenetic Protein: A Cohort of 108 Patients with >2 Years Follow-Up. World Neurosurg..

[21] Hamilton D.K., Smith J.S., Reames D.L., Williams B.J., Shaffrey C. (2010). Use of recombinant human bone morphogenetic protein-2 as an adjunct for instrumented posterior arthrodesis in the occipital cervical region: An analysis of safety, efficacy, and dosing. J. Craniovertebral Junction Spine.

[22] Haque A., Price A.V., Sklar F.H., Swift D.M., Weprin B.E., Sacco D.J. (2009). Screw fixation of the upper cervical spine in the pediatric population. J. Neurosurg. Pediatr..

[23] Hood B., Hamilton D.K., Smith J.S., Dididze M., Shaffrey C., Levi A.D. (2014). The use of allograft and recombinant human bone morphogenetic protein for instrumented atlantoaxial fusions. World Neurosurg..

[24] Savage J.G., Fulkerson D.H., Sen A.N., Thomas J.G., Jea A. (2014). Fixation with C-2 laminar screws in occipitocervical or C1–2 constructs in children 5 years of age or younger: A series of 18 patients. J. Neurosurg. Pediatr..

[25] Sayama C., Hadley C., Monaco G.N., Sen A., Brayton A., Briceño V., Tran B.H., Ryan S.L., Luerssen T.G., Fulkerson D. (2015). The efficacy of routine use of recombinant human bone morphogenetic protein–2 in occipitocervical and atlantoaxial fusions of the pediatric spine: A minimum of 12 months’ follow-up with computed tomography. J. Neurosurg. Pediatr..

[26] Siemionow K., Hansdorfer M., Mardjetko S., Janusz P. (2017). Complications in Adult Patients with Down Syndrome Undergoing Cervical Spine Surgery Using Current Instrumentation Techniques and rhBMP-2: A Long-Term Follow-Up. J. Neurol. Surg. A Cent. Eur. Neurosurg..

[27] Traynelis V.C., Fontes R.B.V., Abode-Iyamah K.O., Cox E.M., Greenlee J.D. (2021). Posterior fusion for fragility type 2 odontoid fractures. J. Neurosurg. Spine.

[28] Yan L., Chang Z., He B., Liu T., Wang X., Guo H., Hao D. (2014). Efficacy of rhBMP-2 versus iliac crest bone graft for posterior C1–C2 fusion in patients older than 60 years. Orthopedics.

[29] Lenke L.G., Betz R.R., Harms J., Bridwell K.H., Clements D.H., Lowe T.G., Blanke K. (2001). Adolescent idiopathic scoliosis: A new classification to determine extent of spinal arthrodesis. J. Bone Jt. Surg. Am..

[30] Ishida W., Ramhmdani S., Xia Y., Kosztowski T.A., Xu R., Choi J., Ramos R.D.l.G., Elder B.D., Theodore N., Gokaslan Z.L. (2019). Use of Recombinant Human Bone Morphogenetic Protein-2 at the C1–C2 Lateral Articulation without Posterior Structural Bone Graft in Posterior Atlantoaxial Fusion in Adult Patients. World Neurosurg..

[31] Brodell D.W., Jain A., Elfar J.C., Mesfin A. (2015). National trends in the management of central cord syndrome: An analysis of 16,134 patients. Spine J..

[32] France J.C., Schuster J.M., Moran K., Dettori J.R. (2015). Iliac Crest Bone Graft in Lumbar Fusion: The Effectiveness and Safety Compared with Local Bone Graft, and Graft Site Morbidity Comparing a Single-Incision Midline Approach with a Two-Incision Traditional Approach. Glob. Spine J..

[33] Xu S., Wang G., Yang J., Zhang S., Song Y., Wang Q. (2022). Anterior debridement, bone grafting and fixation for cervical spine tuberculosis: An iliac bone graft versus a structural manubrium graft. BMC Musculoskelet. Disord..

[34] Hofstetter C.P., Hofer A.S., Levi A.D. (2016). Exploratory meta-analysis on dose-related efficacy and morbidity of bone morphogenetic protein in spinal arthrodesis surgery. J. Neurosurg. Spine.

[35] De Stefano F.A., Elarjani T., Burks J.D., Burks S.S., Levi A.D. (2021). Dose Adjustment Associated Complications of Bone Morphogenetic Protein: A Longitudinal Assessment. World Neurosurg..

[36] Weinberg D.S., Eoh J.H., Manz W.J., Fakunle O.P., Dawes A.M., Park E.T., Rhee J.M. (2022). Off-label usage of RhBMP-2 in posterior cervical fusion is not associated with early increased complication rate and has similar clinical outcomes. Spine J..

[37] Crawford C.H.I., Carreon L.Y.M., McGinnis M.D., Campbell M.J., Glassman S.D. (2009). Perioperative complications of recombinant human bone morphogenetic protein-2 on an absorbable collagen sponge versus iliac crest bone graft for posterior cervical arthrodesis. Spine.

[38] Iyer S., Kim H.J., Bao H., Smith J.S., Gupta M., Albert T.J., Protopsaltis T.S., Mundis G.M., Passias P., Neuman B.J. (2018). The Posterior Use of BMP-2 in Cervical Deformity Surgery Does Not Result in Increased Early Complications: A Prospective Multicenter Study. Glob. Spine J..

[39] Joaquim A.F., Osorio J.A., Riew K.D. (2019). Occipitocervical Fixation: General Considerations and Surgical Technique. Glob. Spine J..

[40] Chen Q., Brahimaj B.C., Khanna R., Kerolus M.G., Tan L.A., David B.T., Fessler R.G. (2020). Posterior atlantoaxial fusion: A comprehensive review of surgical techniques and relevant vascular anomalies. J. Spine Surg..

[41] Ghostine S.S., E Kaloostian P., Ordookhanian C., Kaloostian S., Zarrini P., Kim T., Scibelli S., Clark-Schoeb S.J., Samudrala S., Lauryssen C. (2017). Improving C1–C2 Complex Fusion Rates: An Alternate Approach. Cureus.

